# Pharmacovigilance program to monitor adverse reactions of recombinant streptokinase in acute myocardial infarction

**DOI:** 10.1186/1472-6904-5-5

**Published:** 2005-11-02

**Authors:** Blas Y Betancourt, María A Marrero-Miragaya, Giset Jiménez-López, Carmen Valenzuela-Silva, Elizeth García-Iglesias, Francisco Hernández-Bernal, Francisco Debesa-García, Tania González-López, Leovaldo Alvarez-Falcón, Pedro A López-Saura, the Cuban National Network of Pharmacoepidemiology

**Affiliations:** 1 Clinical Trials Division, Center for Biological Research, Havana, Cuba; 2 National Center for Clinical Trials Coordination, Havana, Cuba; 3 National Coordinating Unit of Pharmacovigilance, Centre for the Development of Pharmacoepidemiology, Minister of Heath, Havana, Cuba; 4 A list of the National Network of Pharmacoepidemiology participating investigators appears in the Appendix

## Abstract

**Background:**

Streptokinase (SK) is an effective fibrinolytic agent for the treatment of acute myocardial infarction (AMI). The objective of the present study was to assess the adverse drug reactions (ADRs) associated with intravenous recombinant SK in patients with AMI in routine clinical practice.

**Methods:**

A national, prospective and spontaneous reporting-based pharmacovigilance program was conducted in Cuba. Patient demographics, suspected ADR description, elements to define causality, and outcomes were documented and analyzed.

**Results:**

A total of 1496 suspected ADRs identified in 792 patients out of the 1660 (47.7 %) prescriptions reported in the program, were received from July 1995 to July 2002. Most of the patients (71.3%) were male, 67.2% were white and mean age was 61.6 ± 13.0 years. The mean time interval between the onset of symptoms and the start of the SK infusion was 4.9 ± 3.7 h. The most frequently reported ADRs were hypotension, arrhythmias, chills, tremors, vomiting, nauseas, allergy, bleeding and fever. ADR severity was 38% mild, 38% moderate, 10% severe, and 4% very severe. Only 3 patients with hemorrhagic stroke were reported. Seventy-two patients died in-hospital mainly because of cardiac causes associated with the patient's underlying clinical condition. Mortality was 3 times more likely in patients suffering arrhythmias than in those without this event (odds ratio 3.1, 95% CI: 1.8 to 5.1). Most of the reported ADRs were classified as possibly or probably associated with the study medication.

**Conclusion:**

Recombinant SK was associated with a similar post-marketing safety profile to those suggested in previous clinical trials.

## Background

Streptokinase (SK) is an indirect fibrinolytic agent that interacts with plasminogen, forming an active complex with protease activity that converts plasminogen to plasmin [1]. SK efficacy with regard to mortality reduction in patients with acute myocardial infarction (AMI) has been demonstrated in large, placebo-controlled trials [2,3].

A SK produced by recombinant DNA techniques (rSK) has been evaluated previously in clinical trials in AMI patients. The first studies suggested that rSK produced the same benefits that were described for natural SK [4,5]. These results were further supported by the Thrombolysis with Recombinant Streptokinase in Acute Myocardial Infarct (TERIMA)-1 trial, a multicenter, randomized, comparative study of rSK vs. natural SK in 224 patients [6]. Both SKs behaved similarly regarding coronary patency at 8 days after thrombolysis and the changes induced on fibrinogen, fibrinogen-degradation-products, and thrombin time. They were also similar with respect to anti-SK antibodies titers and their anti-SK neutralizing activities [7]. The effect on AMI patients' in-hospital mortality was evaluated in a 2923-patients, multicenter, open, phase IV, clinical study (TERIMA-2) [8]. In-hospital 10.4% mortality was found which represented a 4% absolute and a 28.3% relative lethality reduction as compared to a survey made before rSK treatment was introduced.

Clinical trials are usually conducted on a limited number of patients with restrict selection and handling criteria and thus only the most frequent reactions are identified. Postmarketing monitoring is an important procedure to detect some reactions that can become apparent only when the drug is used in a large and varied population. Therefore, an active surveillance program provides a vital service to the heath care system to identify and assess early warning signals, and when appropriate, to take preventive actions to minimize the deleterious effects of drugs.

An adequate safety profile for rSK was observed in clinical trials but it was unknown whether similar results would be found in routine clinical practice. This concern was particularly important for rSK given its nature as a fibrinolytic drug, the potential antigenic capacity due to its bacterial origin, the novelty of the product production process, and its use in emergency situations. Therefore, the further extension of this treatment in Cuba was monitored through an appropriate pharmacovigilance program with the aim to assess rSK safety in AMI patients in routine practice. This paper reports the analysis of data collected after 7 years of such surveillance system implementation.

## Methods

A national, prospective and spontaneous reporting-based pharmacovigilance study was conducted in Cuba. This study was extended to the 14 provinces throughout the different levels of the healthcare system, including municipal, provincial, and clinical-surgical hospitals as well as third-level specialized cardiology units. An adverse drug reaction (ADR) report form was designed. It explicitly asked for the most commonly rSK-associated ADRs and also requested for other reactions. The Clinical Trials Division of the Center for Biological Research and the National Network of Pharmacoepidemiology (NNP) were responsible for the distribution to the hospital services, gathering and quality assessment of ADR report forms. This network is coordinated by the Centre for the Development of Pharmacoepidemiology and its National Coordinating Unit of Pharmacovigilance, as previously described [9]. Since 1994 Cuba is member of the WHO International Drug Monitoring Program.

The rSK (Heberkinasa, Heber Biotec, Havana) studied in this pharmacovigilance system is indicated in AMI as soon as possible, before 12 hours of symptoms onset, in patients without contraindications for thrombolysis. The recommended dose is 1 500 000 IU during 1 hour, through a peripheral vein infusion. Suspected ADRs were identified by physicians through a direct patient evaluation during and after SK administration, as part of the AMI patient assessment. Patient demographics, time between symptom onset and SK infusion, concomitant therapy, outcome (if death and cause of death) and ADR description, including duration, severity, treatment and need for discontinuation, were documented.

An ADR was defined as any noxious, unintended, and undesired effect of a drug that was observed at doses usually administered therapeutically in humans. The medical terminology such as signs and symptoms definition as well as disease or syndrome diagnostic criteria was left to the attending physicians' judgment so that it could reflect routine clinical practice. A temporal or possible association was sufficient for a report to be made.

A qualitative assessment was used to classify the causal relationship as definite, probable, possible or doubtful [10]. According to this method, a reaction was classified as definite if (1) followed a reasonable temporal sequence after drug administration; (2) followed a known pattern of response to the suspected drug; (3) could not be explained by concurrent disease or other drugs; and (4) was confirmed by improvement upon removal of the drug and by reappearance on rechallenge. It was considered as probable if it had the criteria (1), (2), (3) and was confirmed on suspension of the drug but not on rechallenge. A reaction was defined as possible if followed a reasonable time sequence to administration of the drug, but could also be explained by concurrent disease or other drugs. Finally, a reaction that was more likely related to factors other than the suspected drug was classified as doubtful.

The severity of ADRs was classified in four levels: (1) mild if no therapy was necessary, (2) moderate if needed specific treatment, (3) severe when hospitalization or its prolongation was required, and (4) very severe if a reaction was potentially life-threatening or contributed to patient's death.

The causality assessment, as well as the severity analysis, were undertaken by members of the NNP taking into account the reporting physician's criteria, and were further reviewed by the responsible investigators.

The study was sanctioned by the Scientific Committee of the Center for Biological Research. As the study did not involve any intervention apart from the usual medical procedures, and confidentiality of the subjects was maintained, ethics approval and patients' informed consent were not required. The analysis of all ADRs was reported to the National Regulatory Authority.

### Data management and statistical analyses

Databases were double-entered and validated on Microsoft Visual FoxPro version 5.0 and then imported into SPSS version 11.5 for further analysis. Continuous variables are given as means ± standard deviations (SD), whereas discrete variables are given as frequency distributions. Univariate analyses were performed to identify the variables that could influence on adverse reactions and mortality. The analyses were assessed by the χ^2 ^or Fisher's exact tests, depending on the minimum expected values of the tables. A logistic regression analysis was done with variables that resulted statistically significant. The level of significance chosen was p = 0.05.

## Results

A total of 1660 notifications of SK prescriptions was received from July 1995 to July 2002 from 50 hospitals. Of them, 792 (47.7%) reported suspected ADRs. The baseline characteristics of the patients are shown in Table 1. Most of the subjects (71.3%) were male, 67.2% were white and the mean age was approximately 62 years. Age was gender-related: women were 4.4 (95% confidence interval [CI]: 2.9 to 5.9) years older. The mean time interval between the onset of symptoms and the start of the SK infusion was 4.9 ± 3.7 hours. Concomitant acetylsalicylic acid was administered in 81% of the patients.

**Table 1:** Baseline characteristics of the patients (N = 1660)

**Characteristic**	n (% of total)*
Gender	
Male	1183 (71.3%)
Female	430 (25.9%)
Not specified	47 (2.8%)
Ethnicity	
White	1116 (67.2%)
Mestizo	300 (18.1%)
Black	154 (9.3%)
Chinese	8 (0.5%)
Not specified	82 (4.9%)
Age (yr) total (183 not specified)	61.6 ± 13.0
Onset-infusion time (hours)	4.9 ± 3.7
Previous SK	
< 1 year	5 (0.3%)
> 1 year	9 (0.5%)
Concomitant therapy	
Aspirin	1341 (80.8%)
Beta-blockers	943 (56.8%)
Nitrosorbide	258 (15.5%)
Lidocaine	66 (3.9%)
Pentaerythritol tetranitrate	45 (2.7%)
Others	641 (38.6%)

*Continuous variables are presented as Mean ± SD

We found 1496 suspected ADRs identified in 792 patients (1.9 ADRs per patient). The most frequent rSK-related ADRs were hypotension, arrhythmias, chills, tremors, vomiting, and nauseas (Table 2). Allergy was recorded in 47 patients, 34 of them (72%) only with skin rash. Hemorrhage was notified only in 43 patients. ADRs present in less than 5 patients each, included dizziness, dyspnea, arterial hypertension, unspecified pain, paleness, rubor, headache, diarrhea and others.

**Table 2:** Suspected adverse reactions to streptokinase

**ADR**	**n**	%*
Hypotension	285	36.0
Arrhythmias	281	35.5
Chills	212	26.8
Tremors	197	24.9
Vomiting	187	23.6
Nauseas	103	13.0
Allergy	47	5.9
Bleeding	43	5.4
Fever	41	5.2
Lumbar pain	10	1.3
Sweating	9	1.1
Muscular cramp	5	0.6
Others	76	9.6

* % of total patients with ADR, n = 792.

The causality assessment showed that 1419 (94.9%) of the total ADRs were classified as possibly associated with the study medication. Probable and doubtful associations were considered in 63 (4.2%) and 6 (0.4%) of suspected reactions, respectively. Definite reactions were only confirmed in one patient with allergic reactions. The information about eight adverse events was not sufficient for a proper causality assessment.

Of the overall number of suspected ADRs, severity was classified as mild in 564 (37.7%), moderate in 573 (38.3%), severe in 149 (10.0%), and 63 (4.2%) were very severe. Severity was not classified in 147 (9.8%) reactions. Considering the maximal severity to which the patient was exposed, 53 (6.7%) subjects had at least one very severe reaction, which was described for hypotension, arrhythmias and hemorrhage. Of the remaining patients, 104 (13.1%) were exposed to at least one severe reaction.

Definitive discontinuation due to ADRs was observed in 54 (6.8% of those with ADRs) patients. Hypotension, arrhythmias and vomiting were the main causes of treatment withdrawal. Hypotension was also the ADR most frequently leading to temporal SK infusion discontinuation, which was required for the adequate control of 27.7% of the patients experiencing this reaction. The appropriate management of this event included infusion rate reduction, infusion discontinuation, Trendelenburg's position, volume expansion and vasopressors.

ADR occurrence was related to the time between symptoms onset and SK infusion, both in univariate and multivariate analyses. Patients who received SK ≤ 3 hours were more likely to have any ADR as compared to those receiving the infusion after 3 hours (odds ratio 1.5, 95% CI: 1.2 – 1.8). Individual analysis for each suspected ADR showed that arrhythmias were the most time-influenced, being more frequent this event in patients who received SK ≤ 3 h (95% CI: 1.26 – 2.18). The same was true for hypotension (1.08 – 1.85), chills (1.11 – 2.08), and vomiting (1.13 – 2.18). Multivariate analyses confirmed these associations for arrhythmias and hypotension, but for chills and vomiting none of the models obtained were reliable.

Out of the 1660 patients reported, 72 (4.3%) died. Death causes are summarized in Table 3. Cardiac causes (pump failure, arrhythmias, wall rupture), associated mainly with the patient's clinical state, were the most frequent (79.2%). Hemorrhagic stroke was reported as fatal in 3 cases and was the only cause of death that could be directly explained by the use of the study medication.

**Table 3:** Causes of death

**Cause**	**n**	**% of total deaths**
**Cardiac causes**		
Pump failure	32	44.4
Arrhythmias	15	20.8
Wall rupture	10	13.9
**Non cardiac causes**		
Hemorrhagic stroke	3	4.2
Pulmonary embolism	1	1.4
Hypoxic encephalopathy	1	1.4
Sepsis	1	1.4
**Unknown**	9	12.5
**Total**	72	100

Univariate analyses with baseline variables showed that mortality was directly related to older age (p = 1.0 × 10^-7^) and female gender (p = 1.7 × 10^-4^). Patients recorded as dead were 10.1 (95% CI: 7.0 to 13.2) years older than survivors. None of the logistic regression models for the multivariate analysis of the effect of age and gender on mortality was adequate, probably due to the small number of deaths in the sample. Arrhythmias was the only suspected ADR significantly related with death (p = 4.2 × 10^-5^). Mortality was 3 times more probable in patients suffering arrhythmias than in those without this event (odds ratio 3.1, 95% CI: 1.8 to 5.1).

## Discussion

This pharmacovigilance study was performed to further investigate the rSK safety profile in the treatment of AMI patients in clinical practice. Although a national, spontaneous reporting scheme for monitoring all market drugs exists in Cuba, rSK surveillance operated as a separate national system. Using a different ADR report form, this system provided specific information about a thrombolytic drug that otherwise was not possible to obtain (e.g. symptom-infusion time) and also contributed to encourage the number of reports. Results of this study showed a post-marketing safety profile for rSK similar to those suggested in previous clinical trials.

Spontaneous reporting is the most widely used method for pharmacovigilance, but is associated with considerable under-reporting rate [11]. In fact 29,753 rSK doses were distributed throughout the country during the period studied (Heber Biotech, personal communication). Apart from other minor rSK uses, this represents approximately 5.6% of the number of doses used throughout the country in AMI patients. This figure is similar to those showed in other spontaneous reporting schemes [12]. Various possible reasons for not reporting have been identified, including uncertainty as to whether the reaction was caused by a drug, trivial or well known ADRs, unawareness of the need to report ADRs, not enough time and the thought that it is too bureaucratic [13]. All of the above mentioned factors could have influenced the under-reporting observed in this study. In accordance with other spontaneous reporting systems [11], it was not possible to estimate ADRs incidence rates because the numerator was affected by under-reporting and the accurate number of subjects treated (denominator) was unknown.

Time between symptoms and infusion is a critical variable for success of thrombolysis [2,3]. Since the results obtained up to 1995 in the national extension study [8], more experience with the procedures and organizational measures in the heath care system could have improved the time to thrombolysis. However, in the present study this time did not differ significantly from the previous period. This was probably influenced by the under-reporting and do not reflect what actually happened in practice. Currently, the implementation of new organizational actions for the nationwide extension of thrombolysis to the pre-hospital level will surely have a greater impact on this aspect.

Some deviations from the approved indications were observed in routine clinical practice. Although rSK has been approved for routine use within 12 h, 18 patients were reported as receiving the drug after 12 h of symptoms onset. On other hand, the proportions of patients that received aspirin and beta blockers were smaller than previously reported in TERIMA-2 study, where a significant benefit was demonstrated for both drugs.

The most frequent suspected ADRs associated to rSK therapy were hypotension and arrhythmias. This is consistent with the safety profile observed in previous clinical trials. Both events can also be explained by AMI physiopathology and sometimes by other concomitant drugs, so it was difficult to establish a casual relationship with the study medication and all were classified as possible. Hypotension has been described as a transient event that can be related to SK infusion rate [14,15]. Thus, it is recommended to reduce the infusion rate as its initial approach. Arrhythmias have been considered as reperfusion signs after thrombolytic therapy [16], but are also important complications in the course of AMI. Despite the use of thrombolytic therapy, in-hospital ventricular arrhythmias have been associated with higher 30-day and 1-year mortality rates [17,18]. Patients treated earlier after coronary occlusion, when risk is greatest, are more likely to develop ventricular fibrillation [19], which can explain why arrhythmias were more frequent in patients who received SK ≤ 3 h in this study.

Bleeding was notified in 43 patients, of whom only 8 were considered as serious. Hemorrhagic stroke, which is the most severe bleeding complication of fibrinolytic therapy, was reported only in 3 patients (all fatal). This is consistent with the low hemorrhagic stroke rate observed previously in the TERIMA-2 trial (0.3%).

Because SK is an antigenic bacterial protein, physicians need to be aware that some patients experience allergic reactions. Following rSK administration, a significant increase of anti-SK antibodies binding and neutralization titers persisting for 6 months or more have been described [7]. Therefore, concern about SK re-administration exists since it is possible that these antibodies cause allergic reactions or neutralize a further SK dose with the consequent ineffectiveness. Interestingly, 14 patients (5 < 1 year) with antecedent of exposure to SK were reported in this pharmacovigilance and none of them experienced allergic reactions nor account for in-hospital death.

According to the definitions used in this study, at least 19.8% of patients with ADR had a serious one (sum of severe and very severe ADRs). This relatively high proportion is a tendency of spontaneous reporting schemes, since under-reporting involves mainly the less severe and the well known effects [11] so that severe reactions are more likely reported. However, few patients withdrew due to ADRs, emphasizing that prompt recognition and appropriate symptoms handling result in adequate treatment compliance. The in-hospital lethality rate was low but under-reporting could also affect the interpretation of this variable.

Definite reaction assessment was difficult because SK is administered once and rechallenge information is usually not available. Also, many of the SK associated reactions can be explained by concurrent diseases or other drugs as well. Ideally, both, the causality assessment and severity analysis should have been performed by two individuals at the investigation site, separately and then agreement determined. This was not done for practical reasons, since there was only one NNP member at each hospital and thus the evaluation had to be done by this person considering the criteria from the reporting physician, with the subsequent bias derived from application of subjective scales.

Recombinant tissue plasminogen activator (tPA) or its derivatives are recommended by ACC/AHA guidelines to be used in patients who present early after onset of chest pain or symptoms and in those with previous administration of streptokinase and at low risk of intracranial hemorrhage [20]. However, this product is much more expensive than SK and not all countries can afford its general use, especially those with a general health reimbursement policy. Furthermore, a meta-analysis of large trials comparing SK vs. tPA did not demonstrate any clear differences in net clinical outcome because the beneficial cardiovascular effects of tPA is abated by an excess of hemorrhagic stroke [21].

The favorable safety profile achieved with rSK in this and previous studies, together with its demonstrated efficacy and its adequate cost, justifies the extension of this treatment in clinical practice as a reperfusion therapy for AMI patients. Recent reports about deviations from quality of different SK preparations [22,23], raise the importance of a stringent regulation for this product. These reports should be backed up by clinical data [24]. Therefore, results from previous clinical trials and this pharmacovigilance study, along with an appropriate quality control system [25], support the recognized impact of Cuban rSK on AMI mortality in the country.

As in other countries, ischemic heart disease is the major cause of death in Cuba (18.8% of all deaths in 2004; 135.4 per 100000 inhabitants) [26]. The possibility to extend thrombolytic therapy with a safe homemade product is an example of how local biotechnology can influence on one of the main public health problems with a favorable cost/benefit balance, not possible in a developing country with an imported drug, which would had meant a significant economic burden for the public health system.

## Conclusion

In this study, no new signals of previously unknown adverse events were reported, and the nature and severity of the reported events were consistent with information known about rSK from clinical trials. New directions about thrombolysis are emerging in the country, including the use of a new albumin-free formulation [27,28], pre-hospital extension of the procedure, and new indications approval. Therefore, the continuity of this pharmacovigilance would be of inestimable value for safety monitoring of the product.

## Appendix

The National Network of Pharmacoepidemiology participating investigators: Dr. Frank Ravelo González, Dra. Déborah Rodríguez Piñeiro, Dr. Juan C. Mesa Hernández, Lic. Diana R. Fernández Ruiz, Lic. Susana Gómez Pentón, Dr. Rubén Escalante Guardarramos, Lic. Yaneisi C. Perdomo Espinosa, Dr. Ramón Suarez Ramírez, Dra Zaida Herrera López, Lic. Lídice Pérez Incola, Dra. Arlette Linares Borges, Dra. Ismary Alfonso Orta.

## Competing interests

Authors BYB, CVS, EGI, TGL, LAF, and PLS are employees of the Center for Biological Research, which is part of the Center for Genetic Engineering and Biotechnology, Havana network, where recombinant streptokinase is produced. MAMM, FHB were members of the same staff when this work was done. The rest of the authors have no competing interests at all.

## Pre-publication history

The pre-publication history for this paper can be accessed here:


